# Self-Reported Snoring Is Associated with Dyslipidemia, High Total Cholesterol, and High Low-Density Lipoprotein Cholesterol in Obesity: A Cross-Sectional Study from a Rural Area of China

**DOI:** 10.3390/ijerph14010086

**Published:** 2017-01-17

**Authors:** Naijin Zhang, Yintao Chen, Shuang Chen, Pengyu Jia, Xiaofan Guo, Guozhe Sun, Yingxian Sun

**Affiliations:** Department of Cardiology, the First Hospital of China Medical University, Shenyang 110001, China; naijinzhang1989@126.com (N.Z.); chenyintao1990@126.com (Y.C.); loscs@126.com (S.C.); jpydtc1993129@163.com (P.J.); guoxiaofan1986@foxmail.com (X.G.); gzhsun66@163.com (G.S.)

**Keywords:** self-reported snoring, dyslipidemia, high TC, high LDL-C

## Abstract

Studies to explore the relationship between self-reported snoring and dyslipidemia, especially high total cholesterol (TC) and high low-density lipoprotein cholesterol (LDL-C), in the general population are still lacking. Our study was designed to examine whether self-reported snoring is significantly associated with dyslipidemia and ascertain the effects of different snoring intensities on dyslipidemia. There were 10,139 participants in our study. After adjustment for all confounding factors, self-reported snoring (OR = 1.207; *p* = 0.003), moderate (OR = 1.229; *p* = 0.015), strong (OR = 1.222; *p* = 0.033), and very strong (OR = 1.467; *p* = 0.012) snoring intensity, but not low (OR = 1.110; *p* = 0.224) snoring intensity, were significantly associated with dyslipidemia among adults with BMI (body mass index) ≥ 25 kg/m^2^. In addition, self-reported snoring was significantly associated with high TC (OR = 1.167; *p* = 0.048) and high LDL-C (OR = 1.228; *p* = 0.044), rather than low HDL-C (OR = 1.171; *p* = 0.057) and high triglyceride (TG) (OR = 1.110; *p* = 0.141). In conclusion, adults with BMI ≥ 25 kg/m^2^ and who experience snoring, especially moderate, strong, and very strong intensity levels of snoring, should be on the alert regarding the possibility of dyslipidemia, especially high LDL-C and high TC.

## 1. Introduction

Dyslipidemia, which is characterized by high total cholesterol (TC), high triglyceride (TG), low high-density lipoprotein cholesterol (HDL-C), and high low-density lipoprotein cholesterol (LDL-C), is an important disease that is harmful to human health [1]. Previous studies have found that dyslipidemia has a close correlation with the development of atherosclerosis [2], cirrhosis [3], and especially cardiovascular disease (CVD) [4]. Recent studies have found that controlling dyslipidemia can significantly reduce the incidence of CVD [5]. Now, dyslipidemia has a high prevalence in China, and almost one third of the population from developed countries were detected to have dyslipidemia [4,6]. A Chinese National and Health Survey showed that the prevalence of dyslipidemia among the Chinese population was 18.6% (in 2002) [7], however, our present study showed that the prevalence of dyslipidemia has reached 36.3% (in 2012). Therefore, it is highly important to prevent dyslipidemia by identifying its risk factors.

Previous studies have found that self-reported snoring, one of the important manifestations of sleep disorder, was significantly associated with CVD and its risk factors [8,9,10,11,12]. However, as far as we know, studies to explore the relationship between self-reported snoring and dyslipidemia, especially high TC and high LDL-C, in the general population are still lacking. Additionally, previous studies have always focused on the relationship between snoring frequency and CVD and its risk factors, which showed that the risk of CVD and its risk factors were aggravated following an increase in the frequency of snoring [8,9,10,11,12]. However, our previous study found that the risk of cardiovascular outcomes was aggravated when the intensity level of snoring increased [13]. As far as we know, whether different intensity levels of snoring have different effects on dyslipidemia is still unknown.

Therefore, this study is firstly designed to ascertain whether self-reported snoring is significantly associated with dyslipidemia. Additionally, it aims to estimate the effects of different snoring intensity levels on dyslipidemia. Finally, it intends to make clear that self-reported snoring is significantly associated with which kind of dyslipidemia.

## 2. Materials and Methods

### 2.1. Study Population

This cross-sectional survey was conducted from January 2012 to August 2013 in Liaoning Province, located in Northeast China. A representative sample of individuals aged ≥35 years was selected to characterize the prevalence, incidence, and natural history of cardiovascular risk factors in rural areas of Liaoning Province. The study adopted a multi-stage, stratified, random-cluster sampling scheme. First, three counties (Dawa, Zhangwu, and Liaoyang County) were selected randomly from the rural areas of Liaoning province. Next, one town was randomly selected from each of the three counties. Finally, 26 rural villages in three towns were randomly selected for inclusion in the study. Participants who were pregnant, or had a malignant tumor or mental disorder were excluded. All the eligible permanent residents aged ≥35 years from each village were invited to attend the study (a total of 14,016 participants). Of those, 11,956 participants agreed and completed the present study and the response rate was 85.3%. The study was approved by the Ethics Committee of China Medical University (Shenyang, China, ethical approved project identification code: 2011-2-2). All procedures were performed in accordance with the ethical standards. Written consent was obtained from all participants after they had been informed of the objectives, benefits, medical items, and confidentiality agreement of personal information. If the participants were illiterate, we obtained the written informed consents from their proxies. In this report, we used baseline data and only participants with a complete set of data regarding the variables analyzed in the study were included, making a final sample size of 10,139.

### 2.2. Serum Analysis and Definitions

Fasting blood samples were collected in the morning after at least 12 h of fasting for all participants. Blood samples were obtained from an antecubital vein and collected in Vacutainer tubes containing ethylenediaminetetraacetic acid (EDTA). Values for fasting plasma glucose (FPG), TC, LDL-C, HDL-C, TG, and other routine blood biochemical indexes were obtained using an autoanalyzer (Olympus, Kobe, Japan). According to the National Cholesterol Education Program-Third Adult Treatment Panel (ATP III) criteria, high TC was defined as TC ≥ 6.21 mmol/L (240 mg/dL); low HDL-C was defined as HDL-C < 1.03 mmol/L (40 mg/dL); high LDL-C was defined as LDL-C ≥ 4.16 mmol/L (160 mg/dL); and high TG was defined as ≥2.26 mmol/L (200 mg/dL) [14]. Dyslipidemia was defined as having at least one high LDL-C, high TC, low HDL-C, or high TG, as this method had been widely used in China [15,16].

### 2.3. Information on Snoring

Self-reported snoring was defined by a structured questionnaire, including the following questions: 1, Do you know, or has someone previously told you that you snore? (yes or no or do not know); and 2, How loud is the sound of your snoring? The loudness was regrouped into the following four categories: (1) low—subjects whose snoring was slightly louder than breathing sounds; (2) moderate—subjects whose snoring was as loud as normal speaking levels; (3) strong—subjects whose snoring was louder than normal speaking levels; (4) very strong—subjects whose snoring was so loud that it could be heard in the next room. These responses were either self-reported or the response of a close relative and “yes” of the responses of Question 1 indicated that the participants had self-reported snoring. This method had been widely used to obtain the information about snoring in our previous study [13], and in other epidemiologic studies [8,9,10,11,12].

### 2.4. Covariate Measurements and Definitions

Data were collected during a single clinical visit by cardiologists and trained nurses using a standard questionnaire by face-to-face interview. Prior to conducting the survey, all eligible investigators were invited to attend a training session and those who obtained a perfect score on the training test were allowed to participate in this study. Additionally, the investigators received further instructions and support during data collection.

Data on age, sex, and personal history (coronary artery disease, hypertension, and diabetes) and details about lifestyle (current or past cigarette smoking, alcohol intake, dietary habits, educational status, and physical activity) were obtained through questionnaires.

Self-reported sleep durations were categorized into four groups: ≤7, 7–8, 8–9, and >9 h/d. Physical activity included three categories: low, moderate, and high [17]. Blood pressure (BP) was measured using a standardized automatic electronic sphygmomanometer (HEM-907, Omron, Kyoto, Japan) in the sitting position after resting for at least 5 min, according to the recommended American Heart Association protocol. The average BP value was calculated after three different consecutive measurements. The participants were advised to avoid caffeinated beverages and exercise for at least 30 min before the measurement. During the measurement, each participant was seated with their tested arm supported at the level of the heart. According to the Seventh Report of the Joint National Committee on Prevention, Detection, Evaluation, and Treatment of High Blood Pressure (JNC 7), hypertension is defined as systolic blood pressure (SBP) 140 mmHg or greater, diastolic blood pressure (DBP) 90 mmHg or greater, and/or use of antihypertensive medications [18].

### 2.5. Statistical Analysis

Descriptive statistics were calculated for all the variables, including continuous variables (reported as mean values and standard deviations) and categorical variables (reported as numbers and percentages). The differences between non-snoring, low, moderate, strong, and very strong snoring intensity groups were evaluated using ANOVA (LSD) for continuous data and Chi-square test for categorical data. According to participants with BMI (body mass index) < 25 kg/m^2^ or BMI ≥ 25 kg/m^2^, multivariate logistic regression analyses were used to identify the association between self-reported snoring, low, moderate, strong, and very strong snoring intensity and dyslipidemia, high TC, high LDL-C, low HDL-C, and high TG, using odds ratios (ORs) and 95% confidence intervals (CIs). Adjustments for potential confounders were as follows: Model 1 = unadjusted; Model 2 = adjusted for age, gender, education, current smoking status, current drinking status, physical activity, sleep duration, waist circumference, systolic blood pressure, diastolic blood pressure, and diabetes. All the statistical analyses were performed using SPSS version 22.0 software (SPSS Inc., Chicago, IL, USA) and *p*-values less than 0.05 were considered statistically significant.

## 3. Results

### 3.1. Baseline Description of Participants with Different Self-Reported Snoring Intensity

Among the 10,139 participants, 5864 showed non-snoring, and 1412, 1450, 1059, and 354 showed low, moderate, strong, and very strong snoring intensity, respectively. Table 1 showed that the prevalence of dyslipidemia in non-snorers was 32.0% and showed a trend of rising in snorers with increasing levels of snoring intensity as represented by 38.7% in the low group, 41.8% in the moderate group, 45.3% in the strong group, and 48.9% in the very strong group. The prevalence of high LDL-C, low HDL-C, high TG, and high TC had the same trend. The prevalence of high LDL-C was 6.2% in non-snorers, 8.4% in the low snoring intensity group, 9.7% in the moderate snoring intensity group, 9.5% in the strong snoring intensity group, and 8.8% in the very strong snoring intensity group. The prevalence of low HDL-C was 11.3% in non-snorers, 14.7% in the low snoring intensity group, 15.0% in the moderate snoring intensity group, 15.3% in the strong snoring intensity group, and 18.9% in the very strong snoring intensity group. The prevalence of high TG was 14.5% in non-snorers, 18.6% in the low snoring intensity group, 14.6% in the moderate snoring intensity group, 26.4% in the strong snoring intensity group, and 26.6% in the very strong snoring intensity group. The prevalence of high TC was 14.6% in non-snorers, 18.6% in the low snoring intensity group, 19.2% in the moderate snoring intensity group, 19.8% in the strong snoring intensity group, and 18.4% in the very strong snoring intensity group. In addition, with the increase in snoring intensity, the participants were older and had relatively higher prevalence of male gender, current smoking status, current drinking status, diabetes, and higher levels of diastolic blood pressure, systolic blood pressure, fasting plasma glucose, sleep duration, waist circumference (WC), BMI, and lower levels of educational status (*p* < 0.05). However, levels of physical activity were not shown to have a significant difference among the groups.

### 3.2. The Relationship between Dyslipidemia and Self-Reported Snoring, Self-Reported Snoring Intensity According to Participants with BMI < 25 kg/m^2^ or BMI ≥ 25 kg/m^2^

Table 2 showed the multivariable logistic regression analysis of the relationship between self-reported snoring, self-reported snoring intensity, and dyslipidemia according to participants with BMI < 25 kg/m^2^ or BMI ≥ 25 kg/m^2^. Results showed that in the unadjusted BMI < 25 kg/m^2^ group model 1, self-reported snoring (OR = 1.299, 95% CI: 1.150–1.468; *p* < 0.001), strong snoring intensity (OR = 1.492, 95% CI: 1.202–1.851; *p* < 0.001), and very strong snoring intensity (OR = 1.455, 95% CI: 1.017–2.080; *p* = 0.040), but not low snoring intensity (OR = 1.254, 95% CI: 0.951–1.497; *p* = 0.072) and moderate snoring intensity (OR = 1.197, 95% CI: 0.992–1.444; *p* = 0.061), were significantly associated with dyslipidemia. In BMI < 25 kg/m^2^ group model 2, where we adjusted for age, gender, education, current smoking status, current drinking status, physical activity, sleep duration, waist circumference, systolic blood pressure, diastolic blood pressure, and diabetes, self-reported snoring (OR = 1.119, 95% CI: 0.984–1.272; *p* = 0.087), strong snoring intensity (OR = 1.212, 95% CI: 0.967–1.520; *p* = 0.096), and very strong snoring intensity (OR = 1.071, 95% CI: 0.734–1.561; *p* = 0.722) were no longer significantly associated with dyslipidemia. However, results showed that in the unadjusted BMI ≥ 25 kg/m^2^ group model 1, self-reported snoring (OR, 1.374, 95% CI: 1.222–1.546; *p* < 0.001), moderate snoring intensity (OR, 1.377, 95% CI: 1.172–1.618; *p* < 0.001), strong snoring intensity (OR, 1.460, 95% CI: 1.223–1.742; *p* < 0.001), and very strong snoring intensity (OR, 1.896, 95% CI: 1.424–2.524; *p* < 0.001), but not low snoring intensity (OR, 1.178, 95% CI: 0.993–1.398; *p* = 0.060), were significantly associated with dyslipidemia. In addition, after adjusting for age, gender, education, current smoking status, current drinking status, physical activity, sleep duration, waist circumference, systolic blood pressure, diastolic blood pressure, and diabetes in BMI ≥ 25 kg/m^2^ group model 2, we found that self-reported snoring (OR, 1.207, 95% CI: 1.067–1.365; *p* = 0.003), moderate snoring intensity (OR, 1.229, 95% CI: 1.040–1.452; *p* = 0.015), strong snoring intensity (OR, 1.222, 95% CI: 1.016–1.470; *p* = 0.033), and very strong snoring intensity (OR, 1.467, 95% CI: 1.090–1.975; *p* = 0.012) were consistently significantly associated with dyslipidemia.

### 3.3. The Relationship between Each Kind of Dyslipidemia and Self-Reported Snoring According to Participants with BMI < 25 kg/m^2^ or BMI ≥ 25 kg/m^2^

Table 3 showed that according to participants with BMI < 25 kg/m^2^ or BMI ≥ 25 kg/m^2^, we estimated the ORs between each kind of dyslipidemia and self-reported snoring. In the unadjusted BMI < 25 kg/m^2^ group model 1, we found that self-reported snoring was significantly associated with high total cholesterol (OR = 1.285, 95% CI: 1.099–1.503; *p* = 0.002), high low-density lipoprotein cholesterol (OR = 1.355, 95% CI: 1.062–1.730; *p* = 0.015), and high triglyceride levels (OR = 1.301, 95% CI: 1.085–1.559; *p* = 0.004), but not low high-density lipoprotein cholesterol (OR = 1.197, 95% CI: 0.991–1.446; *p* = 0.062). After we adjusted for age, gender, education, current smoking status, current drinking status, physical activity, sleep duration, waist circumference, systolic blood pressure, diastolic blood pressure, and diabetes, we found that self-reported snoring was no longer significantly associated with high total cholesterol (OR = 1.130, 95% CI: 0.960–1.330; *p* = 0.143), high low-density lipoprotein cholesterol (OR = 1.226, 95% CI: 0.953–1.576; *p* = 0.112), and high triglyceride levels (OR = 1.058, 95% CI: 0.874–1.281; *p* = 0.563). However, in the unadjusted BMI ≥ 25 kg/m^2^ group model 1, results showed that self-reported snoring was significantly associated with high total cholesterol (OR = 1.267, 95% CI: 1.093–1.469; *p* = 0.002), high low-density lipoprotein cholesterol (OR = 1.318, 95% CI: 1.086–1.601; *p* = 0.003), high triglyceride (OR = 1.320, 95% CI: 1.156–1.507; *p* < 0.001), and low high-density lipoprotein cholesterol levels (OR = 1.262, 95% CI: 1.082–1.473; *p* = 0.003), respectively. After we adjusted for age, gender, education, current smoking status, current drinking status, physical activity, sleep duration, waist circumference, systolic blood pressure, diastolic blood pressure, and diabetes, we found that self-reported snoring was consistently significantly associated with high total cholesterol (OR = 1.167, 95% CI: 1.001–1.361; *p* = 0.048) and high low-density lipoprotein cholesterol levels (OR = 1.228, 95% CI: 1.006–1.500; *p* = 0.044), rather than low high-density lipoprotein cholesterol (OR = 1.171, 95% CI: 0.996–1.377; *p* = 0.057) and high triglyceride levels (OR = 1.110, 95% CI: 0.966–1.277; *p* = 0.141).

## 4. Discussion

The main finding of our study was that after adjustment for age, gender, education, current smoking status, current drinking status, physical activity, sleep duration, waist circumference, systolic blood pressure, diastolic blood pressure, and diabetes, self-reported snoring and moderate, strong, and very strong snoring intensity, but not low snoring intensity, were significantly associated with dyslipidemia among adults with BMI ≥ 25 kg/m^2^. In addition, we found that the prevalence of dyslipidemia was relatively higher in snorers, and increased with the rise in snoring intensity. Finally, to verify the role that components of dyslipidemia play in this relationship, we adjusted all confounding factors and found that self-reported snoring was significantly associated with high LDL-C and high TC among adults with BMI ≥ 25 kg/m^2^.

Although previous studies have found that self-reported snoring was significantly associated with CVD and its risk factors [8,9,10,11,12,13], relatively little was known regarding dyslipidemia, especially high LDL-C and high TC, in the general population. In this study, we found that after adjustment for all confounding factors, self-reported snoring was an independent risk factor for dyslipidemia, high LDL-C, and high TC among adults with BMI ≥ 25 kg/m^2^. In addition, other studies, which sought to ascertain the role of snoring on various health outcomes, always focused on snoring frequency [8,9,10,11,12]. Our study was the first to explore the relationship between different snoring intensity and dyslipidemia. We found that after adjustment for all confounding factors, moderate, strong, and very strong intensity levels of snoring, rather than low intensity levels of snoring, were significantly associated with dyslipidemia among adults with BMI ≥ 25 kg/m^2^. Our findings provide new insights into the risk factor profile of dyslipidemia and suggest that the population who were BMI ≥ 25 kg/m^2^ and experience snoring, especially moderate, strong and very strong intensity levels of snoring, should be on the alert of the possibility of dyslipidemia, especially high LDL-C and high TC.

Obesity was considered as an independent risk factor for dyslipidemia [19,20], which was consistent in our study. Among adults with BMI ≥ 25 kg/m^2^, we found that after adjustment for all confounding factors, self-reported snoring was significantly associated with dyslipidemia. However, among adults with BMI < 25 kg/m^2^, after adjustment for all confounding factors, self-reported snoring was no longer significantly associated with dyslipidemia, which suggests that obesity may have played a crucial role in the association between snoring and dyslipidemia. In addition, previous studies showed that diabetes and hypertension were significantly associated with dyslipidemia [21,22,23], which proposition was also verified in our study. Our study found that moderate, strong and very strong intensity levels of snoring, rather than low intensity levels of snoring, were significantly associated with dyslipidemia. One possible explanation was that higher intensity levels of snoring, but not lower intensity levels, can reduce the oxygen saturation indices and then cause platelet aggregation and hemodynamic change, which enhance the risk of dyslipidemia [24,25]. Previous studies found that chronic intermittent hypoxia, a manifestation of obstructive sleep apnea (OSA), which can cause the generation of reactive oxygen species, stearoyl coenzyme A desaturase 1, and sympathetic nervous system function, was significantly associated with dyslipidemia [26,27,28]. Therefore, OSA may be another explanation why self-reported snoring was significantly associated with dyslipidemia. Thirdly, inflammation, which was a pathological state of snoring [29], was considered as another possible explanation behind the relationship between dyslipidemia and snoring [30,31].

Previous studies found that there was a close relationship between snoring and metabolic syndrome. Troxel et al. reported that loud snoring was significantly associated with the development of the metabolic syndrome and its components, including low HDL cholesterol [32]. Shin et al. found that snoring was linearly associated with metabolic syndrome and metabolic syndrome components including high blood pressure, high waist circumference, and high fasting glucose, but not low HDL cholesterol and high TG [12]. Our study also found that snoring was not significantly associated with low HDL cholesterol and high TG after adjusting for all those confounding factors. However, as for the other two important kinds of dyslipidemia, high LDL cholesterol and high TC, which were excluded from considerations regarding metabolic syndrome, were also considered to be important independent risk factors for CVD. Our study was the first to find that self-reported snoring was significantly associated with high LDL-C and high TC in BMI ≥ 25 kg/m^2^ population.

Despite the strength of our study, some limitations still remain. Firstly, our study is a cross-sectional study, which is unable to distinguish between cause and effect. As a result, we propose to verify our findings through relevant cohort studies. In addition, the present study was aimed only at a Chinese population. Therefore, our results may not be applicable to the population from other ethnic and regional groups.

## 5. Conclusions

Our study showed that self-reported snoring at moderate, strong, and very strong snoring intensity levels, but not low snoring intensity were significantly associated with dyslipidemia among adults with BMI ≥ 25 kg/m^2^. Among the different kinds of dyslipidemia, self-reported snoring was significantly associated with high LDL-C and high TC levels.

## Figures and Tables

**Table 1:** Baseline description of participants with different self-reported snoring intensity.

Variables	Non-Snoring	Low Intensity	Moderate Intensity	Strong Intensity	Very Strong Intensity	*p*-Value
Mean ± SD	*N* = 5864	*N* = 1412	*N* = 1450	*N* = 1059	*N* = 354	
Dyslipidemia (%)	1875 (32.0)	547 (38.7)	606 (41.8)	480 (45.3)	173 (48.9)	<0.001
High LDL-C (%)	362 (6.2)	119 (8.4)	141 (9.7)	101 (9.5)	31 (8.8)	<0.001
Low HDL-C (%)	660 (11.3)	207 (14.7)	218 (15.0)	162 (15.3)	67 (18.9)	<0.001
High TG (%)	849 (14.5)	263 (18.6)	287 (14.6)	280 (26.4)	94 (26.6)	<0.001
High TC (%)	857 (14.6)	262 (18.6)	279 (19.2)	210 (19.8)	65 (18.4)	<0.001
Age (year)	53.3 ± 11.0	53.7 ± 10.0	54.2 ± 9.8	55.6 ± 9.8	55.6 ± 9.5	<0.001
Male gender (%)	2475 (42.2)	699 (49.5)	725 (50.0)	574 (54.2)	218 (61.6)	<0.001
Educational status (%)						0.006
Primary school or below	2882 (49.1)	668 (47.3)	744 (51.3)	572 (54.0)	198 (55.9)	
Middle school	2448 (41.7)	606 (42.9)	570 (39.3)	386 (36.4)	124 (35.0)	
High school or above	534 (9.1)	138 (9.8)	136 (9.4)	101 (9.5)	32 (9.0)	
Physical activity (%)						0.287
Light	1699 (29.0)	431 (30.5)	432 (29.8)	308 (29.1)	90 (25.4)	
Moderate	3852 (65.7)	890 (63.0)	932 (64.3)	688 (65.0)	237 (66.9)	
Severe	313 (5.3)	91 (6.5)	86 (5.9)	63 (5.9)	27 (7.6)	
Current smoking status (%)	1916 (32.7)	528 (37.4)	545 (37.6)	442 (41.7)	157 (44.4)	<0.001
Current drinking status (%)	1155 (19.7)	364 (25.8)	365 (25.2)	311 (29.4)	99 (28.0)	<0.001
Sleep duration (h/d)	7.2 ± 1.7	7.3 ± 1.6	7.2 ± 1.7	7.3 ± 1.8	7.6 ± 1.8	0.004
SBP (mmHg)	138.9 ± 22.8	142.3 ± 22.6	145.6 ± 23.4	147.4 ± 23.1	146.4 ± 25.2	<0.001
DBP (mmHg)	80.6 ± 11.1	82.9 ± 11.8	84.0 ± 12.0	84.9 ± 11.8	85.0 ± 12.6	<0.001
FPG (mmol/L)	5.8 ± 1.5	6.0 ± 1.7	6.0 ± 1.7	6.0 ± 1.7	6.2 ± 1.9	<0.001
Diabetes (%)	503 (8.6)	154 (10.9)	174 (12.0)	150 (14.2)	55 (15.5)	<0.001
BMI (kg/m^2^)	24.1 ± 3.4	25.1 ± 3.5	25.8 ± 3.7	26.2 ± 4.0	26.4 ± 4.0	<0.001
WC (cm)	80.3 ± 9.2	83.1 ± 9.5	85.3 ± 9.9	86.6 ± 9.7	88.6 ± 10.2	<0.001

The differences between non-snoring, low, moderate, strong, and very strong snoring intensity groups were evaluated using ANOVA (LSD) for continuous data and Chi-square test for categorical data. Abbreviations: BMI, body mass index; WC, waist circumference; DBP, diastolic blood pressure; SBP, systolic blood pressure; HDL-C, high-density lipoprotein cholesterol; LDL-C, low-density lipoprotein cholesterol; TC, total cholesterol; TG, triacylglycerol; FPG, fasting plasma glucose.

**Table 2:** Multivariable logistic regression analysis of the relationship between self-reported snoring, self-reported snoring intensity, and dyslipidemia according to participants with BMI < 25 kg/m^2^ or BMI ≥ 25 kg/m^2^.

Snore Status	BMI < 25 kg/m^2^				BMI ≥ 25 kg/m^2^			
	Model 1	*p*-Value	Model 2	*p*-Value	Model 1	*p*-Value	Model 2	*p*-Value
	OR (95% CI)		OR (95% CI)		OR (95% CI)		OR (95% CI)	
**Self-reported snoring**								
Non-snorers	1.000 (reference)		1.000 (reference)		1.000 (reference)		1.000 (reference)	
Snorers	1.299 (1.150–1.468)	<0.001	1.119 (0.984–1.272)	0.087	1.374 (1.222–1.546)	<0.001	1.207 (1.067–1.365)	0.003
**Self-reported snoring intensity**								
Non-snorers	1.000 (reference)		1.000 (reference)		1.000 (reference)		1.000 (reference)	
Low	1.254 (0.951–1.497)	0.072	1.152 (0.959–1.383)	0.130	1.178 (0.993–1.398)	0.060	1.110 (0.931–1.324)	0.244
Moderate	1.197 (0.992–1.444)	0.061	1.034 (0.850–1.257)	0.741	1.377 (1.172–1.618)	<0.001	1.229 (1.040–1.452)	0.015
Strong	1.492 (1.202–1.851)	<0.001	1.212 (0.967–1.520)	0.096	1.460 (1.223–1.742)	<0.001	1.222 (1.016–1.470)	0.033
Very strong	1.455 (1.017–2.080)	0.040	1.071 (0.734–1.561)	0.722	1.896 (1.424–2.524)	<0.001	1.467 (1.090–1.975)	0.012

Model 1: unadjusted. Model 2: adjusted for age, gender, education, current smoking status, current drinking status, physical activity, sleep duration, waist circumference, systolic blood pressure, diastolic blood pressure, and diabetes. CI: confidence interval; OR: Odds ratio.

**Table 3:** Multiple regression analyses for the relationship between self-reported snoring and each kind of dyslipidemia according to participants with BMI < 25 kg/m^2^ or BMI ≥ 25 kg/m^2^.

Four Kinds of Dyslipidemia	Snorers (BMI < 25 kg/m^2^)		Snorers (BMI ≥ 25 kg/m^2^)	
	OR (95% CI)	*p*-Value	OR (95% CI)	*p*-Value
High total cholesterol				
Model 1	1.285 (1.099–1.503)	0.002	1.267 (1.093–1.469)	0.002
Model 2	1.130 (0.960–1.330)	0.143	1.167 (1.001–1.361)	0.048
Low high-density lipoprotein cholesterol				
Model 1	1.197 (0.991–1.446)	0.062	1.262 (1.082–1.473)	0.003
Model 2	1.114 (0.915–1.355)	0.283	1.171 (0.996–1.377)	0.057
High low-density lipoprotein cholesterol				
Model 1	1.355 (1.062–1.730)	0.015	1.318 (1.086–1.601)	0.005
Model 2	1.226 (0.953–1.576)	0.112	1.228 (1.006–1.500)	0.044
High triglyceride				
Model 1	1.301 (1.085–1.559)	0.004	1.320 (1.156–1.507)	<0.001
Model 2	1.058 (0.874–1.281)	0.563	1.110 (0.966–1.277)	0.141

Model 1: unadjusted. Model 2: adjusted for age, gender, education, current smoking status, current drinking status, physical activity, sleep duration, waist circumference, systolic blood pressure, diastolic blood pressure, and diabetes.
